# Cardioprotective effect of sonic hedgehog ligand in pig models of ischemia reperfusion

**DOI:** 10.7150/thno.40461

**Published:** 2020-03-04

**Authors:** Bijan Ghaleh, Jérôme Thireau, Olivier Cazorla, Raffaella Soleti, Valérie Scheuermann, Alain Bizé, Lucien Sambin, François Roubille, Ramaroson Andriantsitohaina, Maria Carmen Martinez, Alain Lacampagne

**Affiliations:** 1 U955-IMRB, Equipe 03, Inserm, UPEC, Ecole Nationale Vétérinaire d'Alfort, Maisons-Alfort, France.; 2 Université de Montpellier, Inserm, CNRS, CHRU Montpellier, Montpellier, France.; 3 SOPAM, U1063, INSERM, UNIV Angers, SFR ICAT, Angers, France.

**Keywords:** myocardial infarction, arrhythmias, Sonic hedgehog

## Abstract

Sonic hedgehog (SHH) signaling pathway is involved in embryonic tissue patterning and development. Our previous work identified, in small rodent model of ischemia reperfusion, SHH as a specific efficient tool to reduce infarct size and subsequent arrhythmias by preventing ventricular repolarization abnormalities. The goal of the present study was to provide a proof of concept of the cardioprotective effect of SHH ligand in a porcine model of acute ischemia.

**Methods**: The antiarrhythmic effect of SHH, either by a recombinant peptide (N-SHH) or shed membrane microparticles harboring SHH ligand (MPs^SHH+^), was evaluated in a first set of pigs following a short (25 min) coronary artery occlusion (CAO) followed by 24 hours-reperfusion (CAR) (Protocol A). The infarct-limiting effect was evaluated on a second set of pigs with 40 min of coronary artery occlusion followed by 24 hours reperfusion (Protocol B). Electrocardiogram (ECG) was recorded and arrhythmia's scores were evaluated. Area at risk and myocardial infarct size were quantified.

**Results**: In protocol A, administration of N-SHH 15 min. after the onset of coronary occlusion significantly reduced the occurrence of ventricular fibrillation compared to control group. Evaluation of arrhythmic score showed that N-SHH treatment significantly reduced the overall occurrence of arrhythmias. In protocol B, massive infarction was observed in control animals. Either N-SHH or MPs^SHH+^ treatment reduced significantly the infarct size with a concomitant increase of salvaged area. The reduction in infarct size was both accompanied by a significant decrease in systemic biomarkers of myocardial injury, i.e., cardiac troponin I and fatty acid-binding protein and an increase of eNOS activation.

**Conclusions**: We show for the first time in a large mammalian model that the activation of the SHH pathway by N-SHH or MPs^SHH+^ offers a potent protection of the heart to ischemia-reperfusion by preventing the reperfusion arrhythmias, reducing the infarct area and the circulating levels of biomarkers for myocardial injury. These data open up potentially theranostic prospects for patients suffering from myocardial infarction to prevent the occurrence of arrhythmias and reduce myocardial tissue damage.

## Introduction

Sonic hedgehog (SHH) belongs to the Hedgehog protein family that is known to contribute to early embryonic patterning and development and to regulate morphogenesis, organogenesis and left-right asymmetry 1**.** Recently, it was proposed that SHH signaling may play a role in the early postnatal mouse cardiac 2 and lung regenerative responses. In adult tissues, SHH activation has been mainly described to modulate the vascular system. For instance, in mice, SHH promotes angiogenesis 3 and participates in the maintenance of the coronary vasculature 4. SHH signaling has also been shown to promote neovascularization during ischemic diseases 5,6.

We have previously reported that activation of SHH pathway either by a recombinant SHH protein (N-SHH) or by shed membrane microparticles (MPs) derived from activated/apoptotic T lymphocytes and harboring SHH (MPs^SHH+^) protects myocardium against ischemia-reperfusion damage in rodent models 7. MPs are plasma membrane-derived vesicles released from cells during activation by agonists, physical or chemical stress. Global composition of MP proteins can be related to stimulus at their origin and to the mother cell type 8-10 and may explain, at least in part, the beneficial or deleterious effects elicited by MPs in physiological or pathological conditions. The cardio-protection that they conferred is mediated by electrophysiological properties modulations directly at the cardiomyocyte level 7. As previously reported in endothelial cells 11,12, activation of SHH induces the phosphatidylinositol 3-kinase (PI3-K)/Akt/nitric oxide (NOS) pathway and nitric oxide (NO) release from the cardiomyocytes 7. SHH-induced NO formation specifically activates the ATP-dependent inward rectifying current (IK_ATP_) independently from ATP depletion. In normal situation, this results in a reduction of the action potential duration (APD) and *in vivo* in a reduction of the QT interval of the ECG. During acute myocardial infarction, SHH activation reduces the QT interval, the risk of arrhythmic events and the infarct size 7. These effects were abolished by cyclopamine, a specific inhibitor of the SHH pathway which binds to the SHH receptor, Smoothened (SMO). Moreover, the incubation of isolated cardiomyocytes with MPs lacking SHH protein-derived from apoptotic T lymphocytes had no effect on cardiomyocytes 7. Other groups also suggested that strategies increasing the SHH signaling pathway may offer useful tools for improving cardiac dysfunction after myocardial infarction in mice 13-15. It should be noted, however, that other groups using isolated perfused mouse heart have shown that SMO activation could be proarrhythmic by lengthening ventricular repolarization 16.

Because of major differences in cardiac electrical properties such as heart rate, APD and shape, between rodent and large mammals 17, the cardioprotective effect of SHH signaling after acute ischemia in pre-clinical models close to human remains to be determined. To this aim, we translated our previous results 17 in a porcine model of ischemia-reperfusion where the antiarrhythmic effect of SHH was evaluated in a first set of pigs following a short period (25 min) of coronary artery occlusion. The infarct size-limiting effect was evaluated on a second set of animals following 40 min of coronary artery occlusion. Our data clearly demonstrate both reduced arrhythmias and infarct size in the porcine model and confirm the potential interest at developing this approach for patients with acute myocardial infarction.

## Methods

The experimental protocol was approved with the local animal ethical committee [ComEth ANSES-ENVA-UPEC agreement 16].

### Experimental protocol

Landrace Large-White crossed pigs (Lebeau, Gambais France) (20-30 kg) were sedated with azaperone (8mg/kg). General anaesthesia was induced with ketamine (15 mg/kg) then, the animals were intubated, and anesthesia was maintained with isoflurane (1.5-2.5 vol%). A left thoracotomy was performed at the fifth intercostal space with use of sterile surgical technique. Fluid-filled Tygon catheters were implanted in the proximal descending thoracic aorta, in the left atrium and in the pulmonary artery (used for drug administration). A balloon occluder was placed around one of the major branches of the left descending coronary artery. All catheters were exteriorized between the scapulae. The incision was closed in layers. Ceftiofur (4 mg/kg, i.m.) and long acting amoxicillin (15 mg/kg every two days, i.m.) were administered during five days after surgery. Fentanyl (patch 100µg/h sc) was administered three days after surgery.

The experiments were conducted 2 weeks after surgery when pigs were healthy and apyretic. All animals were studied in a sling. Animals were anesthetized with zoletil (12.5 mg/kg/h during 25 min) and thiopenthal (50 mg/kg/h during 25 min). Coronary artery occlusion (CAO) was induced by inflating the balloon occluder and was followed by coronary artery reperfusion (CAR) by deflating the balloon occluder. Electrocardiogram (ECG) was recorded and conventional RR, PR intervals and QRS, QTc (Bazett's formula) durations were determined using HEM 4.2 (Notocord, Croissy sur Seine, France).

Pigs were studied in two different protocols. In the one hand (Protocol A), to investigate the antiarrhythmic properties of SHH, CAO was induced in 25 pigs during 25 min followed by 24 h CAR. In case of ventricular fibrillation (both during occlusion and coronary reperfusion), an external shock (100 to 300 J) associated with a chest massage was performed to allow the resumption of spontaneous cardiac activity. In this case, gentian violet was immediately administered for the delineation of the risk zone. The electrophysiological consequences of ischemia/ reperfusion depend on the duration of ischemia, with most reperfusion arrhythmias triggered after short duration of ischemia. Indeed, the presence of numerous surviving muscle fibres in the area at risk provides both anatomical and electrophysiological substrates for re-entry, favouring the occurrence of arrhythmia. In the other hand, to determine the infarct-size limiting properties of SHH, a second protocol (Protocol B) was used with longer duration of ischemia *i.e.* 40 min CAO followed by 24 h CAR to induce large myocardial infarction (n=14 pigs). In both protocols, intra venous administration of 0.9% NaCl or N-SHH (25 μg/kg in 0.9% NaCl) occurred 15 min after the onset of CAO.

In another set of experiments, N-SHH was substituted by MPs^SHH+^ (see below) at the dose of 625 µg of total MP proteins/Kg and animals underwent 40 min CAO and 24 h CAR. As N-SHH, MPs were administered intravenously.

In case of ventricular fibrillation (both during occlusion and coronary reperfusion), an external shock (100 to 300 J) associated with a chest massage was performed to allow the resumption of spontaneous cardiac activity.

### Determination of infarct size

At sacrifice, pigs were re-anesthetized and the balloon occluder was inflated again. Crystal violet solution (0.8%) was administered intravenously to delineate the previously occluded vascular bed (area at risk, AAR). The heart was excised and the left ventricle was cut into slices that were weight and incubated in 1% triphenyltetrazolium chloride (TTC) at 37°C to measure the extent of myocardial lesion. Slices were fixed overnight in 10% formaldehyde and then photographed. Using a computerized planimetric program (Image J, NIH, Bethesda, MD, USA), the AAR, the infarcted and salvaged zones were delimitated and quantitated. The AAR was identified as the non-violet region and was expressed as a percentage of the left ventricle weight. Infarcted area was identified as the TTC negative zone and was expressed either as a percentage of AAR. The viable tissue or salvaged area was the difference between AAR and the infarcted area and was expressed as percentage of AAR.

### Determination of arrhythmia scores

**Figure 1:**
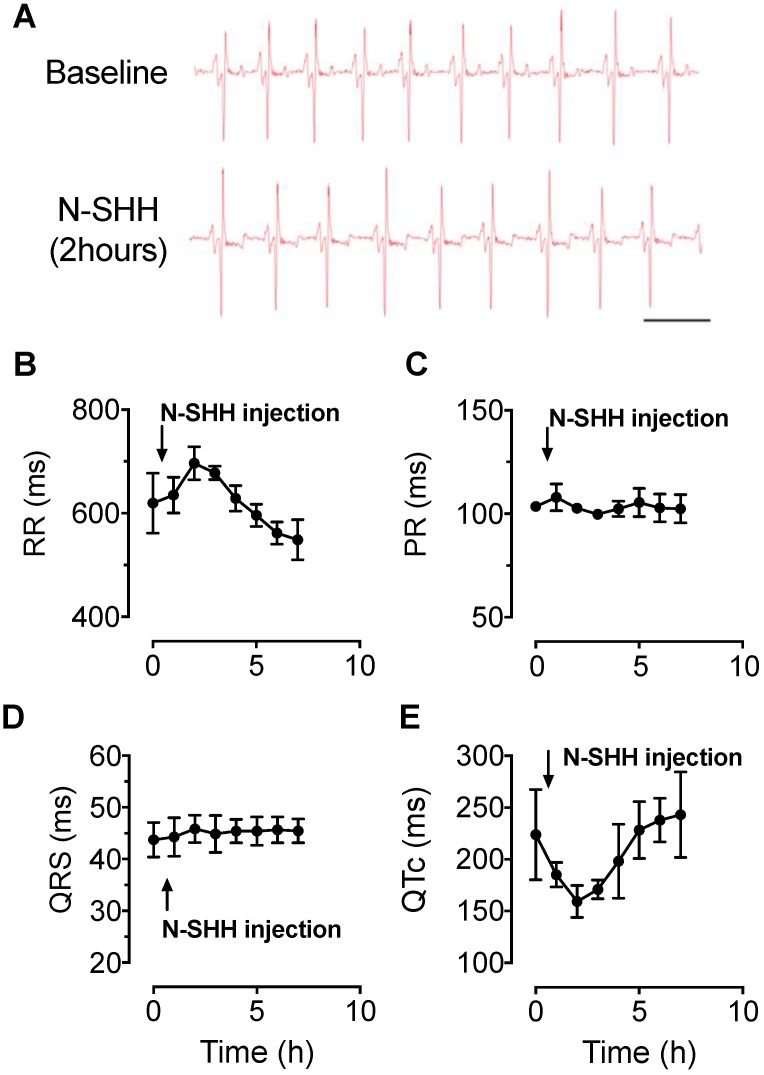
** Electrophysiological effect of SHH in healthy pigs.** ECG parameters were continuously recorded over a period of 7 hours after intravenous injection of N-SHH (25μg / kg) in healthy pigs. (**A**) Representative traces of ECG recorded in a healthy animal before (Top) and 2 hours after injection of N-SHH (Bottom). (**B-E**) Average time course of RR (**B**) PR (**C**), QRS (**D**) and QT_C_ (**E**) intervals (n=3 healthy pigs).

**Figure 2:**
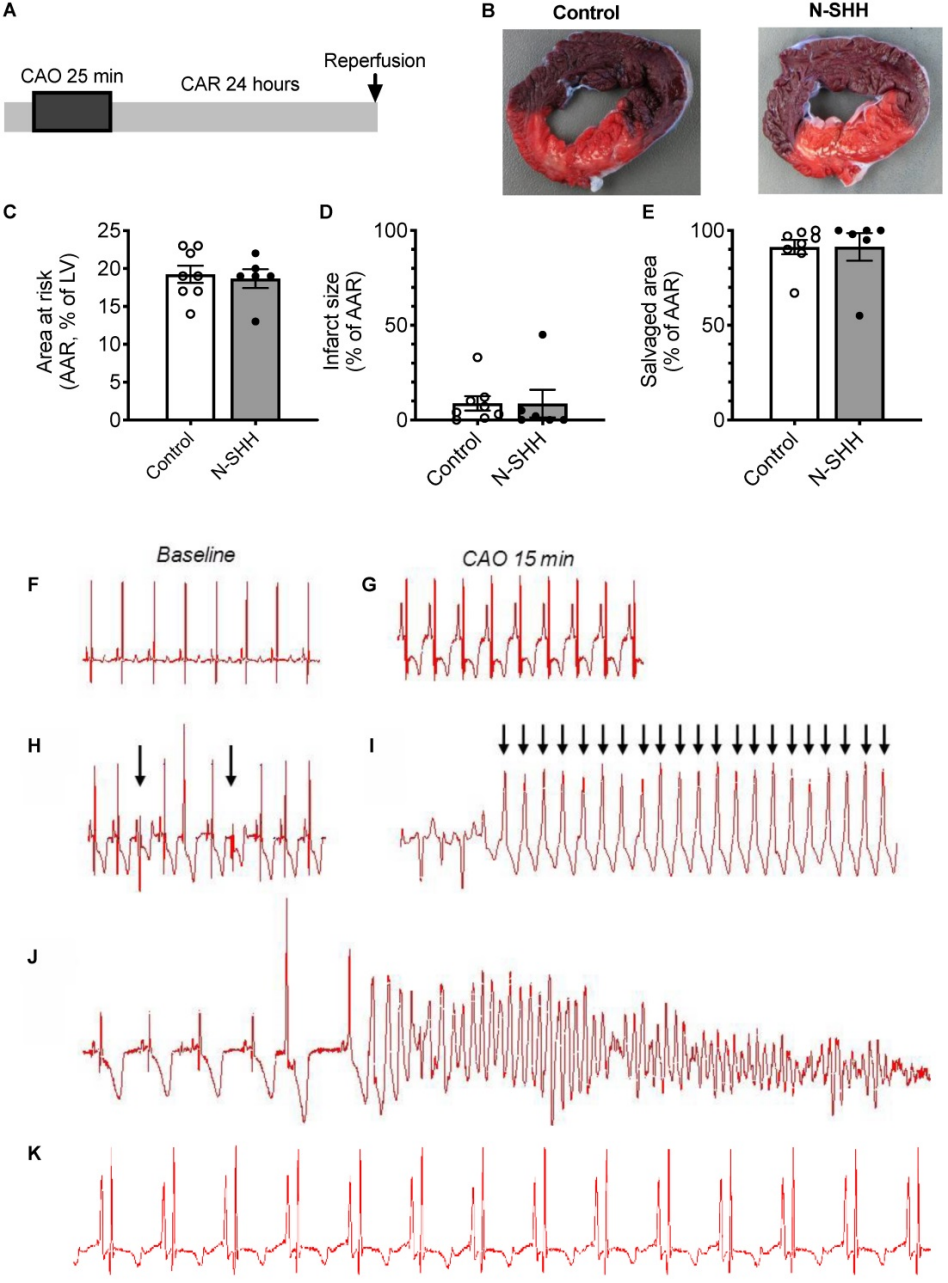
** Arrhythmias induced by short ligation model and low infarct size**. (**A**) Description of the occlusion reperfusion protocol. (**B**) representative picture of the crystal violet/ TTC colored heart, 24 hours after ischemia reperfusion in a non-treated (Control, left panel) and a N-SHH treated (right) pig. All data are summarized in (**C**) Mean area at risk (AAR), (**D**) infarct size and salvage area (n=8 controls, n=6 N-SHH). Representative electrocardiogram recordings in non-treated animals at baseline (**F**) and during coronary artery occlusion (CAO) (**G**) in a pig in normal sinus rhythm. Representative recordings of ventricular premature beats (indicated by arrows, **H**), of ventricular tachycardia (**I**) and of ventricular fibrillations (**J**). **K**/ Representative trace obtained during reperfusion period in I/R animal treated with N-SHH.

The occurrence of arrhythmias was quantitated during CAO and CAR using a scoring system previously reported 18 and adapted from Curtis et Walker 19 according to the Lambeth convention 20. It assigned one score per pig representing the most severe type of arrhythmia observed during CAO and 4 h-CAR. Ventricular premature beats were defined as identifiable premature QRS complexes. Ventricular tachycardia (VT) was defined as a run of four or more ventricular premature beats. Ventricular fibrillation (VF) was defined as a signal for which individual QRS deflections can no longer be distinguished from one other and for which a rate can no longer be measured 20. Baseline ECG recordings are illustrated in Figures 1A and 2F. Arrhythmia scores were assigned as summarized in the Table 1. With the aim to test antiarrhythmic properties of SHH signalling cascade, animal who presented a cumulated score = 0 in both 0-15 min and 15-25 min occlusion phases were excluded of the study, this could suggest a failing in ischemia-induced arrhythmia. In final, 21 animals were further analysed. Some animals triggered VF, which required an electrical chock and chest massage to allow the resumption of spontaneous cardiac activity. In this case, scoring was stopped and done until the VF occurred.

### Western blotting

Fifty µg of lysed proteins from left ventricular samples from ischemic epicardial region were separated by 10% sodium dodecyl sulfate-polyacrylamide gel electrophoresis and transferred on to nitrocellulose membranes. Membranes were then saturated at room temperature for 60 min in TBS-T buffer (200 mM Tris base, 615 mM NaCl pH 7.8 and 1% Tween 20) containing 5% BSA. Membranes were probed overnight at 4°C using antibodies against anti-endothelial NOS (eNOS, BD biosciences, #610297; 1:1000), anti-phospho-eNOS Serine 1177 (BD biosciences, #612392; 1:1000) and anti-GAPDH antibodies (Santa Cruz Biotechnologies, #47724, 1:1000). The membranes were then washed at least three times in Tris buffer solution containing 0.05% Tween 20 and incubated for 1 h at room temperature with the appropriate HRP-conjugated secondary antibody (Amersham Biosciences, Piscataway, NJ). The protein-antibody complexes were detected by ECL-Plus Chemiluminescence kit (Amersham Biosciences) according to the protocol of manufacturer. The relative intensity of the immunoreactive bands was determined by densitometry using Image J software (Rasband, W.S., ImageJ, U. S. National Institutes of Health, Bethesda, Maryland, USA, http://imagej.nih.gov/ij/, 1997-2011). The results were normalized to GAPDH levels and expressed as fold-change over control.

### Isolation of membrane MPs^SHH+^ from CEM T Cells

The human lymphoid CEM T cell line (ATCC, Manassas, VA) was used for microparticles (MPs) production. Cells were seeded at 10^6^ cells/ml and cultured in serum-free X-VIVO 15 medium (Lonza, Walkersville, MD). MPs were produced by pharmacological treatment of CEM T cells as previously described 11,21. Briefly, CEM T cells were treated with phytohemagglutinin (5 µg/mL) for 72 h, and then with phorbol-12-myristate-13 acetate (20 ng/mL) and actinomycin D (0.5 µg/mL) for 24 h. A supernatant was obtained in two centrifugation steps at 750 *g* for 15 min and at 1500 *g* for 5 min to remove cells and large debris. Remaining MP- containing supernatant was subjected at 14,000 *g* for 45 min to pellet MPs. MP pellet was subjected at two series of centrifugations at 14,000 *g* for 45 min. Finally, MP pellet was recovered in 400 µL sterile NaCl (0.9% w/v) 21. Concentration of MPs was determined by measuring MP-associated proteins, using the method of Lowry, with BSA as the standard. SHH carried by MPs was detected by Western blot as previously described 21.

### Chemicals

Recombinant Human Sonic Hedgehog/Shh (C24II) N-Terminus, Carrier Free was purchased from R&D Systems (Bulk 1845-SH/CF, Bio-techne, Lille, France) and administrated at 25 ug/kg as reported previously 7. For assessment of heart damage, we measured pig Fatty Acid Binding Protein (HFABP-9, Life Diagnostics Ltd) and pig cTnI (CTNI-9-HS, Life Diagnostics Ltd) from the serum using ELISA kits, according to the manufacturer protocol. All other chemicals, unless stated, were obtained from Sigma (St. Louis, MO, USA).

### Statistics

Data are expressed as Mean ± standard error of mean (SEM). Results were considered significant with p < 0.05, with analysis of variance (ANOVA) followed by Bonferroni post-test. The risk to trigger VF during reperfusion was assessed between SHH and saline group by the Fisher's exact test and Chi-square test.

## Results

### Effect of SHH on electrocardiogram in healthy pigs

The activation of SHH pathway was first tested in control healthy pigs by the evaluation of N-SHH on the ECG parameters. ECGs were monitored continuously over a period of 7 h (Figure 1A). The administration of N-SHH resulted in a transient decrease in heart rate as observed by the increased RR interval (Figure 1B) and a decrease in the QT interval corrected for heart rate (QTc, Figure 1E), with a maximal effect 2 h after injection. The PR and QRS interval were unchanged (Figure 1C, D). This effect on QTc interval was similar to that previously reported in rats 7. These results indicate that (i) SHH pathway can be activated in pig myocardium and (ii) by analogy with the rat model, this effect is likely to be due to a direct effect of N-SHH on the duration of the ventricular action potential repolarization.

### Antiarrhythmic effect of SHH after acute myocardial infarction

**Figure 3:**
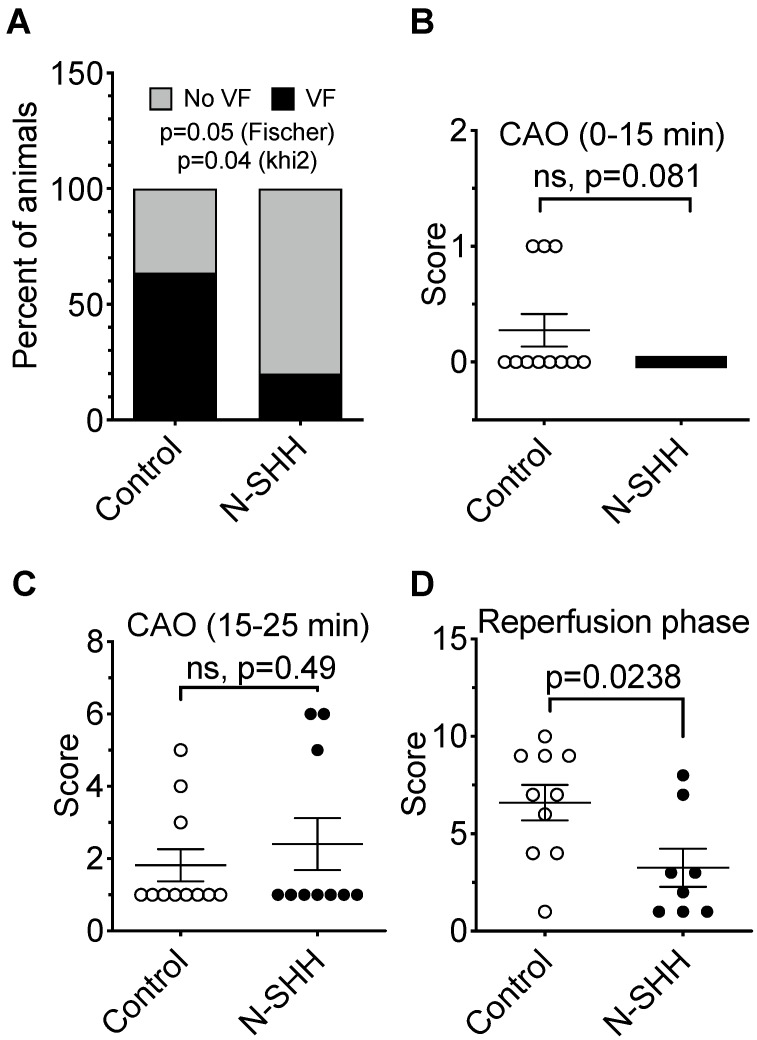
** Antiarrhythmic effect of SHH.** (**A**) Distribution of ventricular fibrillation (VF) during CAR following short-ischemia protocol in non-treated (Control) vs N-SHH treated (N-SHH). N-SHH reduced significantly the occurrence of VF (p=0.05 with a Fischer test and p=0.04 with a Khi2). The overall arrhythmic was estimated according to a score (table 1) during the first 15 minutes of occlusion (**B**), during the last 10 minutes of occlusion (**C**) (n=11 in control and n=10 in SHH group) and during reperfusion (**D**) (n=10 in control and n=8 in SHH group). N-SHH treatment significantly reduced the overall occurrence of arrhythmias (p=0.0238).

To evaluate the beneficial effect of SHH on ventricular arrhythmias following ischemia-reperfusion, we performed a set of experiments where the coronary artery was occluded for only 25 min and N-SHH was injected 15 min after the onset of CAO (Figure 2A). As previously reported by others, short period of ischemia favors the amount of viable tissue and the occurrence of arrhythmic events 22. The size of the AAR was not significantly different between the 2 groups (control, 19±1% vs SHH, 20±1%) (Figure 2B-2C). The infarct size was, as expected, very small and similar between the two groups (9±4% vs 9±7%, in control and N-SHH, respectively) (Figure 2B, 2D). Also, the salvaged area was similar among groups (Figure 2E). At reperfusion, several forms of ventricular arrhythmias were observed such as ventricular premature beats, ventricular tachycardia and ventricular fibrillations when compared with baseline (Figure 2F-J). When considering all animals exhibiting arrhythmic events during CAO, occurrence of ventricular fibrillation during CAR was significantly reduced in N-SHH compared to control group (20%, 2/10 animals *vs* 64%, 7/11 animals respectively, Figure 3A). We also evaluated the antiarrhythmic effect of SHH using a scoring system (Table 1), as previously reported by our group 18. The overall arrhythmias were estimated according to this score during the first 15 min of CAO (Figure 3B), during the last 10 min of CAO (Figure 3C) and during CAR (Figure 3D). N-SHH treatment significantly reduced the overall occurrence of arrhythmias during CAR (p=0.024) without modification during CAO.

### Cardioprotective effect of SHH on massive infarction

**Figure 4:**
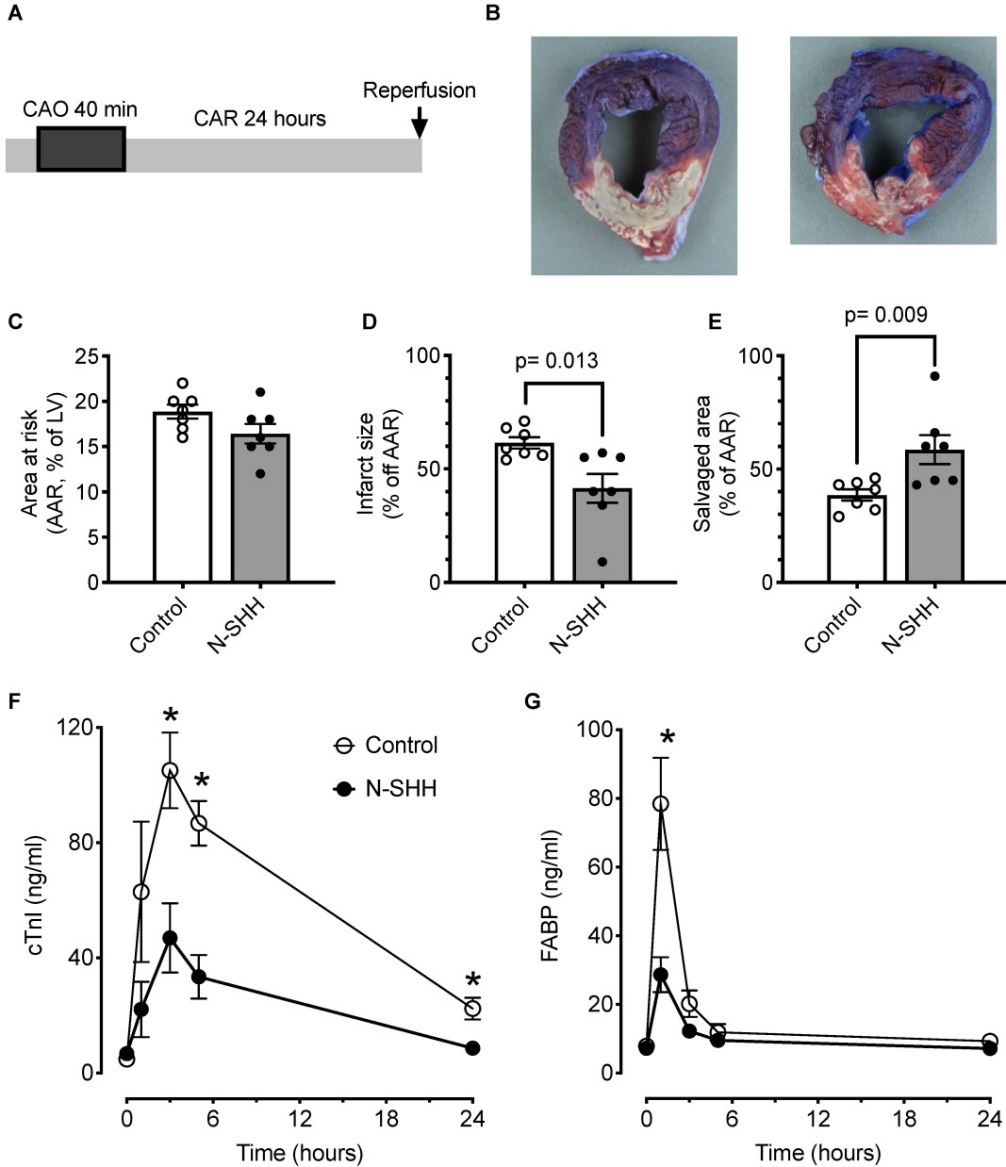
** Cardioprotective effect of SHH**. (**A**) Description of the occlusion reperfusion protocol. (**B**) Representative picture of the crystal violet/TTC colored heart, 24 hours after ischemia reperfusion in non-treated (control, left) and N-SHH treated (right) animals. Note the reduction of the white colored myocardial part in N-SHH compared with control hearts. All data are summarized in (**C**) Mean area at risk (AAR), (**D**) infarct size and (**E**) salvaged area. Time courses of circulating biomarkers of myocardial injury, cardiac troponin I (cTnI, **F**) and fatty acid-binding protein (FABP, **G**). n=7 controls and n=7 N-SHH, *, p<0.05 control *vs* N-SHH.

Cardio-protection of therapeutic strategies on myocardial infarction size is classically explored after a long coronary occlusion, thus animals underwent a coronary occlusion of 40 min followed by 24 h of reperfusion (Figure 4A). In a first set of experiments, the animals received 0.9% NaCl or recombinant N-SHH 15 min after the onset of CAO.

A total of 14 pigs were included in the protocol (n=7 controls, n=7 SHH). The size of the AAR was similar between the 2 groups (control, 19±1% vs SHH, 16±1%) (Figure 4B-C). Under these conditions, the size of the infarct was significantly reduced by 33% thanks to the administration of SHH (control, 61±1% *vs* SHH, 41±6%) (Figure 4B-D). The salvaged area was significantly increased (control, 39±2% *vs* SHH, 59±6%) (Figure 4E). The reduction in infarct size was accompanied by a significant decrease in systemic myocardial damage biomarkers, *i.e.*, cardiac troponin I (cTnI, Figure 4F) and fatty acid-binding protein (FABP, Figure 4G). ECG was recorded and, as expected, exhibited in both group a low occurrence of arrhythmic events. Nevertheless, this analysis also revealed a reduced number of sinus pauses (Figure S1A), number of extra ventricular systoles (Figure S1B) and number of ventricular fibrillations (Figure S1D) without changes in ventricular tachycardia (Figure S1C) within the first 4 h following reperfusion.

**Figure 5:**
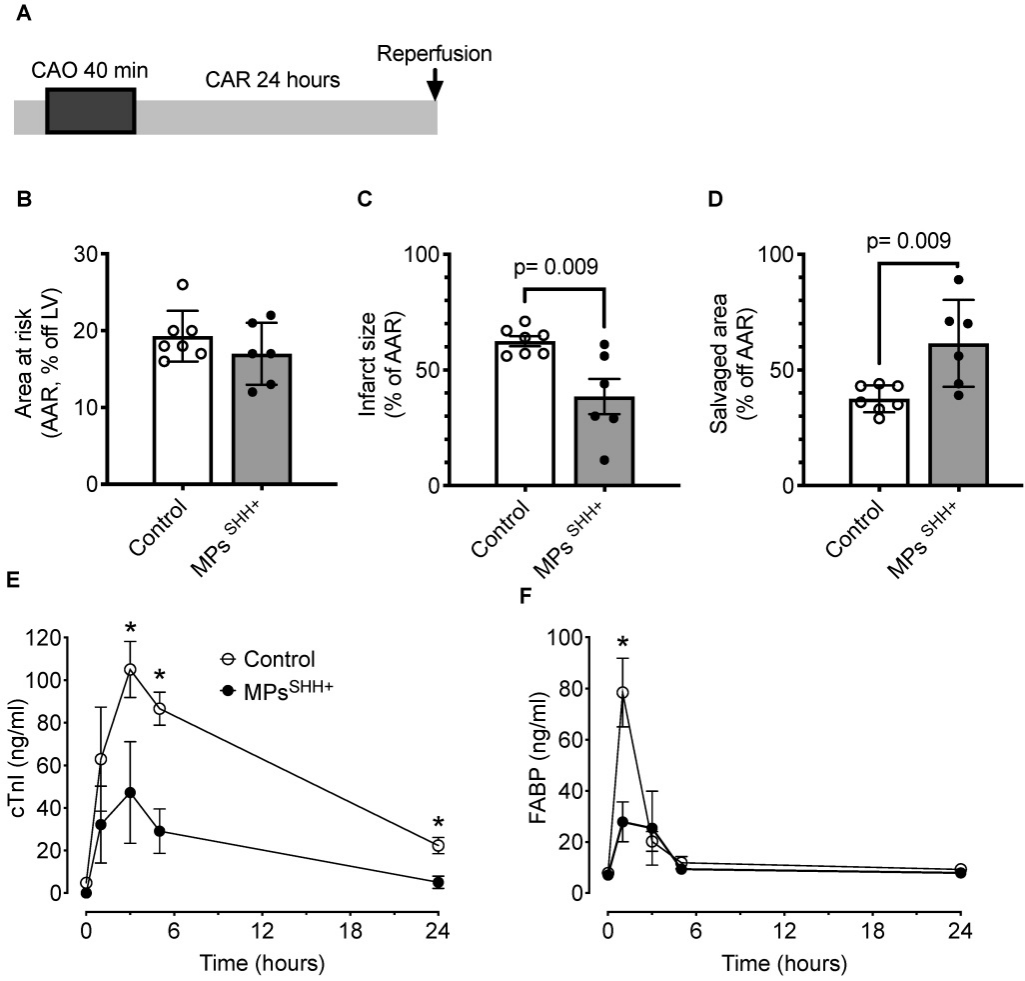
** Cardioprotective effect of MPs^SHH+^**. (**A**) Description of the occlusion reperfusion protocol. (**B**) Mean area at risk, (**C**) Mean infarct size and (**D**) Mean salvaged area. Time courses of circulating biomarkers of myocardial injury, cardiac troponin I (cTnI, **E**) and fatty acid-binding protein (FABP, **F**). n= 7 non-treated (control) and n= 6 MPs^SHH+^ animals, *, p<0.05 MPs^SHH^ *vs* control. The control values used for panel E and F are the same as in figure 4.

An alternative approach to stimulate endogenously SHH pathway is to use MPs^SHH+^ 21. Thus, in a second series of experiments, recombinant N-SHH was substituted by MPs^SHH+^ (n=7 controls and n=6 MPs^SHH+^) (Figure 5A). In these conditions, one could observe after 40 min of occlusion a similar cardioprotection with a significantly reduced infarct size (Control, 62±2% *vs* MPs^SHH+^, 38±8%) (Figure 5B-C) while the salvaged area was significantly increased (Control, 38±2% vs SHH, 62±8%) (Figure 5D). Here, again the reduction in infarct size was accompanied by a significant decrease in circulating cTnI (Figure 5E) and FABP (Figure 5F).

### SHH signaling exerts a cardioprotective effect via NO pathway

**Figure 6:**
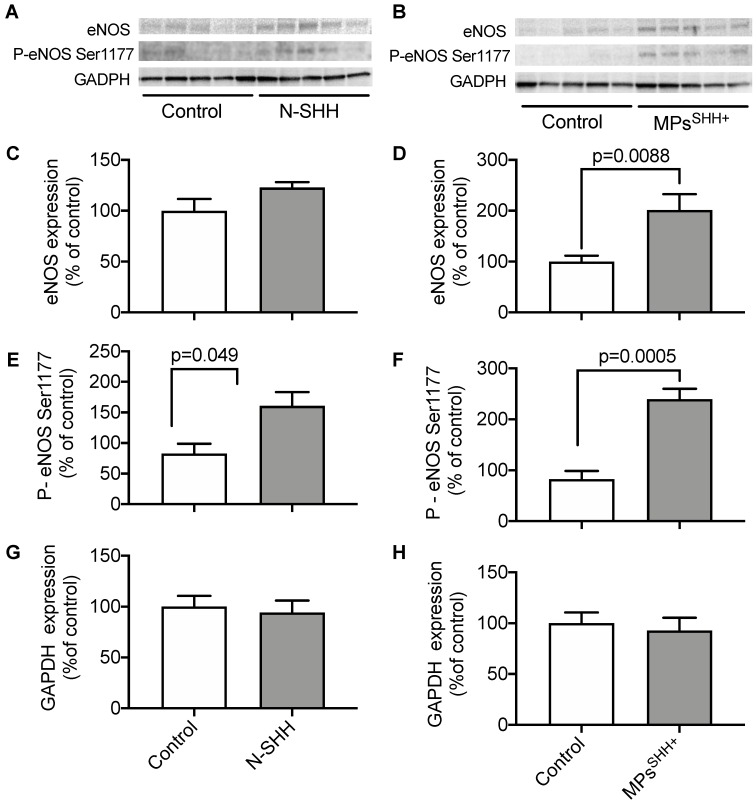
** Effect of N-SHH** and **MPs^SHH+^ treatment on NO pathway after 40min CAO.** Western Blot analyses on ventricular samples in animals submitted to 40 min CAO followed by 24 h CAR. (**A, B**) Representative images showing the expression of endothelial NO-synthase (eNOS), phosphorylation on its activator site (Ser 1177) and reference glyceraldehyde 3-phosphate dehydrogenase (GAPDH) in left ventricular samples from ischemic epicardial region. (**B, C**) Quantification of eNOS expression (**D, E**), activation and (**G, H**) GADPH expression. (n=5 non-treated controls, n=5 N-SHH, n=5 MPs^SHH+^).

By analogy with the signaling pathway identified in rats, we hypothesized that activation of the NO pathway contributes to the cardioprotective effect mediated by N-SHH and MPs^SHH+^. To test this hypothesis, we performed Western Blot analyses on ventricular samples in animals submitted to 40 min CAO followed by 24 h CAR, to assess the level of expression of eNOS and the level of phosphorylated eNOS on Serine 1177 in (Figure 6). The expression of eNOS tended to increase in the presence of N-SHH and significantly increased with MPs^SHH+^ (Figure 6D). More interestingly, eNOS phosphorylation at Serine 1177 by AKT has been shown to increase NO production 23,24. Here, we observed that both N-SHH (Figure 6E) and MPs^SHH+^ (Figure 6F) increased the phosphorylation of eNOS to Serine 1177 suggesting increased production of NO in these tissues.

## Discussion

In the present study, we have established the proof of concept for the cardioprotective and antiarrhythmic effects of SHH pathway during ischemia-reperfusion in a relevant preclinical large animal model. We used a short duration of ischemia (*i.e.* 25 min) to test the antiarrhythmic effect and a long duration (*i.e.* 40 min) to test the infarct size-limiting effect of SHH. We show for the first time in a large mammalian model that the activation of the SHH pathway either by a recombinant protein (N-SHH) or by MPs^SHH+^ offers a protection of the heart against the consequences of ischemia-reperfusion by preventing the reperfusion arrhythmias and reducing the infarct area.

We previously reported in a rat model of ischemia-reperfusion that stimulation of SHH pathway, either by a recombinant peptide or MPs^SHH+^, prior to reperfusion reduces both infarct size and subsequent arrhythmias by preventing ventricular repolarization abnormalities 7. We further demonstrated in the rat model, that the reduction of QTc interval *in vivo* was mediated by NO/cGMP pathway leading to the shortening of ventricular cardiomyocytes APD due to the activation of an inward rectifying potassium current sharing pharmacological and electrophysiological properties with ATP-dependent potassium current. Although it was not investigated at the cellular level in the present study, we speculate, based on the qualitative reduction in QTc that occurs in healthy pigs, that the cardioprotective effect of SHH signaling occurs in pigs via mechanisms similar to those observed in rats 7. This speculation is also supported by the increased in eNOS phosphorylation observed after activation of SHH signaling. This however contrasts with a recent publication indicating that SHH could prolong APD 16. The divergence with previous work about SHH repolarizing effect may be explained by difference in the used-methodology (i.e. *in vivo* vs isolated perfused heart), or by intervention of other effectors of the HH signaling pathway (SMO ligand *vs* SHH ligand) 16. Nevertheless, the effect of N-SHH in control healthy pigs on the QT interval of the ECG is consistent a previous report 7. By analogy, we thus speculate a direct ventricular cardiomyocyte effect of N-SHH inducing a shortening of the action potential accounting for the reduced QT interval. As described in the rat model, such homogeneous reduction of ventricular APD may prevent the myocardium from calcium overload that occurs during ischemia reperfusion 25 and preserve cellular energy balance throughout myocardial territory. Interestingly, both N-SHH and MPs^SHH+^ reduced circulating levels of biomarkers of myocardial injury such as cTnl and FABP. These biomarkers are severely increased in serum of patients with acute myocardial infarction 26,27. In the present study, circulating levels of both biomarkers return to baseline after 24 h of either N-SHH or MPs^SHH+^ treatment suggesting that the cardioprotection exerted by SHH pathway is rapidly efficient, not only in modulating cardiomyocyte electrophysiological properties, but also in reducing precociously myocardial injury. Despite the potential benefit to prevent/limit the reperfusion lesions as in clinics, this does not directly imply an improvement in adverse post-ischemic left ventricular remodeling and in left ventricular function, i.e., major end-points that are required to be met to reduce post-myocardial infarction morbi-mortality. To provide this second proof before clinical translation, another study with animals being followed for several weeks after reperfusion should be performed to evaluate the beneficial effects of SHH on left ventricular function with e.g. echocardiography and pressure-volume loop acquisitions.

The role of NO signaling pathway in cardioprotection is well documented 28,29. SInce, SHH pathway induces NO production both in endothelial cells and cardiomyocytes, this may also contribute to the cardioprotective effect described here. Nevertheless, the involvement of such pathway in a long-term process also needs further investigations. In agreement with our previous studies, both N-SHH and MPs^SHH+^ increased the expression of NO-synthase as well as its activation as observed by the increase of phospho-NO-synthase at the activator site (Serine 1177). Altogether, these data suggest that the increase on NO production into myocardium could participate in the cardioprotective effect associated to SHH.

### Clinical significance

While the rodent model, previously used to evaluate the cardioprotective effect of SHH signaling 7, presents significant differences in term of cardiac physiological and pathological regulations with patients, the pig model used in the present study is extremely similar 17. As a matter of fact, the literature abounds with validated cardioprotective strategy in rodents but not in the large mammal that have failed to reveal protection during clinical investigations 30. Contrasting with rodents or rabbits, large animal models using pigs have the advantage of resembling to humans regarding the size and anatomy of the heart, systemic hemodynamics and particularly heart rate or spatial and temporal development of myocardial infarction 31. Moreover, the choice of two distinct durations of ischemia is also highly relevant since in patients, the delay before reperfusion may significantly vary from one patient to the other. Finally, in Paulis et al 7, the proof of concept was provided by an injection of N-SHH or MPs^SHH+^ prior to the coronary artery occlusion which is not clinically relevant. Here, the administration either of SHH ligand or MPs^SHH^ is performed rather during ischemia.

In case of acute myocardial infarction, the reopening of the artery is mandatory and needed as early as possible in clinical practice 32. The incidence of severe ventricular arrhythmias has declined over recent decades, mainly because of early reperfusion and early use of beta-blockers. However, 6-8% of patients still develop hemodynamically significant severe ventricular arrhythmias following the reperfusion 7. Repetitive electrical cardioversion as a first-line, but also beta-blockers or other antiarrhythmic drugs including amiodarone, lidocaine could be mandatory. The patients with life-threatening ventricular arrhythmias can suddenly die during the early phase of 30 days with a risk of mortality without long-term arrhythmic risks 34. In this context, targeting SHH signaling could then offer a promising cardioprotection both by limiting the infarct size but also by decreasing the early ventricular arrhythmias.

### Study limitations

The use of a large animal model limits considerably the number of experiments that can be easily performed in rodents. In addition, the staining of the myocardium for the evaluation of the risk zone and the size of the infarction limited us from further exploring at the tissue and/or cellular level a potential cascade (i.e. NO production) explaining the cardioprotective effect of SHH. We thus limited here our interpretation to the *in vivo* effect of N-SHH on the QT interval in healthy animals, the increase in eNOS phosphorylation and the analogy with Paulis et al. 7. N-SHH or MPs^SHH+^ were not administered at the onset of reperfusion but 10 to 15 min before reperfusion. To reinforce the clinical relevance, a whole study including a therapeutic window and long-term effects of N-SHH or MPs^SHH+^ on left ventricular remodeling and function would be required.

## Conclusion

The beneficial effects of the activation of the SHH pathway in a porcine model of ischemia reperfusion opens a potentially interesting therapeutic prospective for patients suffering from a myocardial infarction to prevent the occurrence of reperfusion arrhythmias and reduce the infarct size.

## Supplementary Material

Supplementary figure.

## Figures and Tables

**Table 1:** Ventricular arrhythmia score system.

Score	Coronary artery occlusion		Coronary artery reperfusion
	0-15 min	15-25 min	
**0**	< 10 PVT	< 10 PVT	< 10 PVT
**1**	>= 10 PVT	>= 10 PVT	>= 10 PVT
**2**	VT (Duration < 30s)	VT (Duration < 30s)	VT (Duration < 30s)
**3**	VT (Duration > = 30s)	VT (Duration > = 30s)	VT (Duration > = 30s)
**4**			VF starting 15 min after the onset of reperfusion
**5**	VF starting 5 to 15min after the onset of ischemia	VF starting 20 min after the onset of ischemia	VF starting 5 to 15min after the onset of reperfusion
**6**	VF starting 5min after the onset of ischemia	VF starting 15 to 20 min after the onset of ischemia	VF starting 5min after the onset of reperfusion

VT, ventricular tachycardia, VF ventricular fibrillation, PVT premature ventricular beat

## Abbreviations

APD: Action Potential Duration
AAR: area at risk
ECG: Electrocardiogram
QTc: Corrected QT interval
MPs^SHH+^: microparticles harbouring SHH at the surface
