# Supplementary material for: Cardioprotective effect of sonic hedgehog ligand in pig models of ischemia reperfusion
Source: Theranostics. 2020 Mar 4;10(9):4006–16. doi: 10.7150/thno.40461 (PMC7086352; doi:10.7150/thno.40461)
Supplement: Supplementary file 1 — Supplementary figure. [file thnov10p4006s1.pdf]

Supplemental Informations:

Cardioprotective effect of sonic hedgehog ligand in pig models of ischemia reperfusion

Bijan Ghaleh*[1], Jérôme Thireau*[2], Olivier Cazorla[2], Raffaella Soleti[3], Valérie Scheuermann[2], Alain Bizé[1], Lucien Sambin[1], François Roubille[2], Ramaroson Andriantsitohaina[3], Maria Carmen Martinez[£ 3], Alain Lacampagne[£ 2]

*Same contribution

[1] U955-IMRB, Equipe 03, Inserm, UPEC, Ecole Nationale Vétérinaire d'Alfort, Maisons-Alfort, France
[2] Université de Montpellier, Inserm, CNRS, CHRU Montpellier, Montpellier, France
[3] SOPAM, U1063, INSERM, UNIV Angers, SFR ICAT, Angers, France

£ correspondance to :
Dr Alain Lacampagne
Phymedexp, UMR CNRS 9214 – Inserm U1046
Physiologie et Médecine Expérimentale du Cœur et des Muscles
CHU Arnaud de Villeneuve
34295 Montpellier Cedex 05
Phone : + 33 467 415 228
Fax : +33 467 415 242
email: alain.lacampagne@inserm.fr

Dr Maria Carmen Martinez
Inserm U1063
IBS Institut de Biologie en Santé
4 rue Larrey
49933 ANGERS Cedex 9
Phone : + 33 244 688 584
email: carmen.martinez@univ-angers.fr

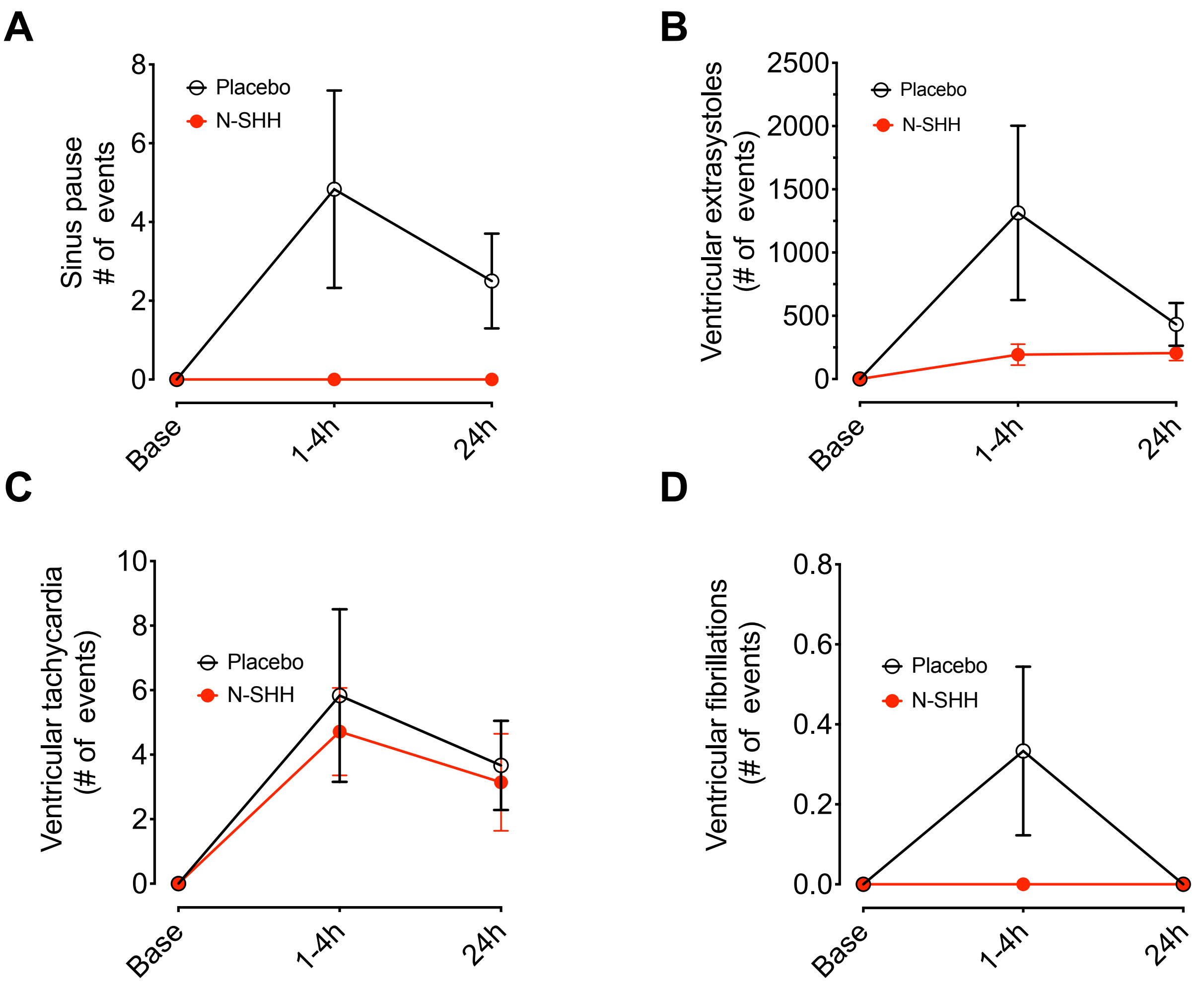

Figure S1: Reperfusion arrhythmias and N-SHH treatment after 40 minutes of coronary artery occlusion. (A) sinus pause, **(B)** ventricular extra-systoles, **(C)** ventricular tachycardia and (**D)** ventricular fibrillation measured before ischemia (base) during 3 hours starting 1 hour after reperfusion (1-4h) and at 24 hours after reperfusion (24h) (n=7 Controls, n=7 SHH).
